# Serum biomarkers in people with chronic low back pain and Modic 1 changes: a case-control study

**DOI:** 10.1038/s41598-019-46508-x

**Published:** 2019-07-10

**Authors:** Margaux Boisson, Didier Borderie, Yves Henrotin, Stéphanie Teboul-Coré, Marie-Martine Lefèvre-Colau, François Rannou, Christelle Nguyen

**Affiliations:** 1 AP-HP, Service de Rééducation et de Réadaptation de l’Appareil Locomoteur et des Pathologies du Rachis, Hôpitaux Universitaires Paris Centre - Groupe Hospitalier Cochin, 75014 Paris, France; 2 Université de Paris, UFR Pharmacie, Sorbonne Paris Cité, 75006 Paris, France; 3 AP-HP, Service de Diagnostic Biologique Automatisé, Hôpitaux Universitaires Paris Centre - Groupe Hospitalier Cochin, 75014 Paris, France; 4 INSERM UMR 1124, Toxicité environnementale, cibles thérapeutiques, signalisation cellulaire, UFR Sciences Fondamentales et Biomédicales, 75006 Paris, France; 5 Bone and Cartilage Research Unit, Arthropôle Liège, University of Liège, Liège, Belgium; 6 Artialis SA, GIGA Tower, CHU Sart-Tilman, 4000 Liège, Belgium; 7 Université de Paris, UFR Médecine, Sorbonne Paris Cité, 75006 Paris, France; 8 INSERM UMR 1153, Centre de Recherche Épidémiologie et Statistique Paris Sorbonne Cité (CRESS), ECaMO Team, 75006 Paris, France; 9 Institut Fédératif de Recherche sur le Handicap, 75013 Paris, France

**Keywords:** Diagnostic markers, Skeleton

## Abstract

We aimed to compare serum biomarkers of inflammation, redox status and cartilage degradation between chronic low back pain (cLBP) patients with and without Modic 1 changes. We used a convenience sample of patients recruited from a single center, case-control study, conducted in a tertiary care center. From December, 2014 to May, 2016, 2,292 patients were consecutively screened, 34 met inclusion criteria and were prospectively enrolled in the present study. Cases (n = 13) were defined as patients with Modic 1 changes detected on MRI and controls (n = 21) as cLBP patients without (Modic 0). To assess serum biomarkers of inflammation, redox status and cartilage degradation, fasting serum samples were collected in a standardized manner and analyzed by immunoassays and spectrophotometry. Mean (95% CI) age was 44.1 (40.0–48.1) years and mean LBP duration was 72.5 (53.0–91.9) months. Serum biomarkers of inflammation (IL-1β, IL-6, IL-8 and TNF-α), redox status (total thiols, advanced oxidation protein products and carbonyl groups) and cartilage degradation (Coll2-1 and Coll2-1NO_2_) did not differ between cLBP patients with and without Modic 1 changes. In summary, we did not find any differences in serum biomarkers between cLBP patients with and without Modic 1 changes. Interpretation is limited by convenience sampling and small sample size.

## Introduction

Non-specific chronic low back pain (cLBP) is a common symptom. It is the leading cause of life years lived with disability in the world^1^. The etiological diagnosis of LBP is a challenge because consistent anatomoclinical correlation is lacking. In the late 1980s, elementary changes of vertebral endplate subchondral bone were described adjacent to degenerative disc disease on MRI (Modic classification)^2^. In a subset of chronic LBP patients, Modic 1 changes on MRI are associated with clinical^3,4^ and laboratory signs^4^. This condition is now refered as active discopathy^5,6^.

Patients with cLBP and active discopathy have inflammatory-like LBP and low grade local and systemic inflammation^5,6^, but do not fulfill the classification criteria for spondyloarthropathies^3^. Consistently, targeting local inflammation with intradiscal anti-inflammatory drugs have positive effects on pain at short term^7–9^. These observations suggest that local inflammation could explain in part active discopathy-related symptoms. However, effects of anti-inflammatory therapies are not sustained, which suggests that aside from inflammation, other elementary molecular pathways may contribute to active discopathy-related symptoms. For example, changes in cartilage remodeling have been described during active discopathy and include decreased expression of chondrogenesis transcription factors such as type 2 collagen and Sox-9^10^. Some authors also reported an increase in oxidative and nitrosative stress markers in patients with active discopathy as compared to those without^11^.

We hypothesized that along with inflammation, other elementary molecular pathways including redox status and cartilage degradation may be activated in patients with cLBP and active discopathy, and aimed to compare serum biomarkers of interest between cLBP patients with and without active discopathy.

## Methods

### Study design

We used a convenience sample of patients with cLBP with or without active discopathy recruited from a previous cross-sectional case-control study performed from December, 2014 to May, 2016, in a single tertiary care center (Rehabilitation Department, Cochin Hospital, Paris, France)^12^. Our study is reported in accordance with the Strengthening The Reporting of OBservational Studies in Epidemiology checklist^13^ (see supplementary material).

### Participants

All patients referred to our department were prospectively and consecutively screened. Patients fulfilling the following inclusion criteria and who agreed to participate were prospectively enrolled in the primary study: male sex, age ≥18 and <65 years, Caucasian, cLBP defined as a daily LBP persisting for ≥3 months, lumbar MRI ≤6 months, lumbar type 1 Modic changes at a single level with preserved intervertebral disc height (intervertebral space narrowing <50%, maximal height of the intervertebral disc visually estimated on mid-sagittal T2-weighted MRI and described as a percentage of the nearest normal above disc height) for cases, and lumbar type 0 Modic changes for controls. Patients were included in the present study if at least one of the serum markers of interest could be assessed. A complete description of the inclusion and exclusion criteria was published^12^.

### Lumbar MRI

A lumbar MRI with T1-, T2- and Short Tau Inversion Recovery (STIR)-weighted sequences ≤6 months were qualitatively assessed by 1 investigator (STC) for presence and level of types 0, 1, 2 and 3 Modic changes for each patient.

### Serum biomarkers

Fasting serum samples were collected at baseline (between 7 and 9 am, before breakfast and after at least a 48 hr-wash-out of non-steroidal anti-inflammatory drugs, smoking and alcohol consumption) and stored at −40 °C. Protein carbonyl groups were detected and quantified by spectrophotometry using 2,4-dinitrophenylhydrazine^14^. The carbonyl content was expressed as nanomoles of carbonyl per milligram of proteins. Thiol determinations were based on the thiol/disulfide reaction of thiol and Ellman’s reagent, 5,5′-dithiobis(2-nitrobenzoic acid)^15^. Advanced oxidation protein products (AOPP) were quantified as described previously^16^. AOPP concentrations were expressed as micromoles per liter (μmol/L) of chloramine T equivalent. Coll2-1, a peptide located in the triple helical part of type 2 collagen molecule, and its nitrated form (Coll2-1NO_2_) were measured by using enzyme-linked immunosorbent assay (ELISA) following the recommendations of the manufacturer (Artialis, Liège, Belgium, www.artialis.com) and cytokines (IL-1β, IL-6, IL-8 and TNF-α) by using the Meso Scale Discovery array technology (MSD, Rockville, USA).

### Statistical methods

For descriptive analyses, qualitative variables were reported with absolute and relative frequencies and quantitative variables with mean (95% CI). Normal distribution of quantitative variables was assessed by the Shapiro-Wilk test. For comparative analyses, serum biomarkers were compared using the Student t-test for normally-distributed variables and using the Mann-Whitney test for non-normally-distributed variables.

### Ethical consideration

The study was carried out in accordance with L.1123-6 article of the French Health Code. The study protocol was approved by the local ethics committee (*Comité consultatif de Protection des Personnes en Recherche Biomédicale d*’*Île*-*de*-*France 1*). Informed written and oral consent was obtained from all participants.

### Sources of funding and role of funders

The study was funded by *Assistance Publique*-*Hôpitaux de Paris* (project n°MERRI-AAP-2017-008). The funding source was not involved in the study design, data collection or interpretation, statistical analysis, manuscript preparation, or the decision to submit the manuscript for publication.

## Results

### Patients

Overall, 2,292 patients were consecutively screened, 37 met inclusion criteria and were prospectively enrolled in the primary study. The full flow diagram was published^12^. Because for 3 patients, serum samples were not sufficient to perform at least 1 laboratory test, serum from 34/37 patients were analysed in the present study. Mean (95% CI) age was 44.1 (40.0–48.1) years and mean LBP duration was 72.5 (53.0–91.9) months (Table 1).

**Table 1:** Participants’ baseline characteristics.

	Control (Modic 0) n = 21	Cases (Modic 1) n = 13	All patients n = 34	p-value*
**Demographic characteristics of patients**				
Age (years), mean (95% CI)	42.0 (37.6; 46.4)	47.4 (38.9; 56.0)	44.1 (40.0; 48.1)	0.23
Body mass index (kg/m^2^), mean (95% CI)	23.4 (22.3; 24.5)	26.3 (24.4; 28.3)	24.5 (23.5; 25.6)	0.01
Currently working (yes), n (%)	17 (81.0)	9 (69.2)	26 (76.5)	0.43
Currently smoking (yes), n (%)	9 (42.9)	6 (46.2)	15 (44.1)	0.85
**Characteristics of chronic low back pain**				
Low back pain duration (months), mean (95% CI)	62.7 (41.4; 84.0)	88.2 (47.9; 128.5)	72.5 (53.0; 91.9)	0.24
Low back pain intensity (0-100 mm VAS), mean (95% CI)	45.5 (34.1; 56.8)	47.6 (35.0; 60.2)	46.3 (38.2; 54.4)	0.79
Quebec score (0-100), mean (95% CI)	30.8 (25.0; 36.5)	37.5 (29.4; 45.5)	33.3 (28.7; 37.9)	0.16
Worst painful moment in the morning or at night and/or morning stiffness (yes), n (%)	7 (33.3)	8 (61.5)	15 (44.1)	0.11
**Current treatments** (**yes**), **n** (**%**)				
Painkillers	14 (66.7)	5 (38.5)	19 (55.9)	0.11
Non-steroidal anti-inflammatory drugs	4 (19.1)	3 (23.1)	7 (20.6)	0.78

No missing data; 95% CI: 95% confidence interval.

*Comparisons between Modic 0 and Modic 1 groups.

Numbers in the brackets are 95% CI, unless otherwise indicated.

### Serum biomarkers of inflammation

Mean concentrations of serum markers of inflammation (IL-1β, IL-6, IL-8 and TNF-α) did not differ between the 2 groups (Table 2).

**Table 2:** Serum biomarkers of inflammation, redox status and cartilage degradation.

Biomarkers, mean (95% CI)	Control (Modic 0) n = 21	Cases (Modic 1) n = 13	All patients n = 34	p-value
Inflammation				
IL-1β (pg/ml)*	0.2 (0.1; 0.3)	0.2 (0.1; 0.3)	0.2 (0.1; 0.3)	0.466
IL-6 (pg/ml)*	0.9 (0.5; 1.2)	0.8 (0.5; 1.1)	0.8 (0.6; 1.0)	0.906
IL-8 (pg/ml)*	53.8 (−1.8; 109.3)	23.3 (0.0; 46.6)	41.8 (8.0; 75.6)	0.796
TNF-α (pg/ml)*	3.2 (2.4; 3.9)	2.8 (2.3; 3.2)	3.0 (2.6; 3.5)	0.869
Redox status				
Total thiols (µM)**^£^	333.7 (309.1; 358.3)	318.8 (271.4; 366.2)	327.8 (305.4; 350.3)	0.554
Advanced oxidation protein products (µM)*^$^	17.5 (5.0; 30.0)	12.8 (6.4; 19.2)	15.5 (8.1; 23.0)	0.246
Carbonyl groups (nmol/mg of proteins)*^#^	1.0 (0.5; 1.5)	1.2 (0.8; 1.7)	1.1 (0.8; 1.4)	0.246
Cartilage degradation				
Col2-1 (nM)**^§^	269.8 (239.2; 300.4)	241.6 (214.4; 268.8)	258.5 (237.7; 279.4)	0.150
Coll2-1NO2 (pg/ml)*°	790.9 (549.8; 1032.0)	830.2 (577.6; 1082.8)	805.8 (638.4; 973.2)	0.472

IL: interleukin; TNF: Tumor Necrosis Factor.

*Comparisons between Modic 0 and Modic 1 groups using the Mann-Whitney test.

**Comparisons between Modic 0 and Modic 1 groups using the Student t-test.

The sample size varied depending on the parameter analysed. All these variations were due to low sample amount.

^£^n = 33 patients (20 controls and 13 cases).

^$^n = 30 patients (18 controls and 12 cases).

^#^n = 32 patients (19 controls and 13 cases).

^§^n = 31 patients (18 controls and 13 cases).

°n = 29 patients (18 controls and 11 cases).

Numbers in the brackets are 95% CI, unless otherwise indicated.

### Serum biomarkers of redox status

Mean concentrations of serum markers of redox status (total thiols, advanced oxidation protein products and carbonyl groups) did not differ between the 2 groups (Table 2).

### Serum biomarkers of cartilage degradation

Mean concentrations of serum markers of cartilage degradation (Coll2-1 and Coll2-1NO_2_) did not differ between the 2 groups (Table 2).

## Discussion

In the present study, we did not find any differences in serum biomarkers of inflammation, redox status and cartilage degradation between cLBP patients with and without active discopathy.

With coefficients of variation ranging from 5 to 10% for spectrophotometric tests and less than 15% for immunoassays, our results are reliable. However, whether the techniques used were sensitive enough to detect systemic low-grade changes in inflammation or redox status in this population remains unknown. Because active discopathy is associated with disc degeneration and because chondral plate degradation and type 2 collagen is found in the *nucleus pulposus* and chondral plates^17^, we anticipated an increase of Coll2-1 and Coll2-1NO_2_ level in the serum of patients with active discopathy^18–20^. The absence of changes in these biomarkers levels was unexpected. The absence of pro-inflammatory cytokines increase in the serum of patients with active discopathy was also surprising. However, consistently with our findings, Capossela and colleagues did not find any differences in the serum levels of various inflammatory mediators including IL-1β, IL-6 and TNF-α between people with LBP and pain-free volunteers^21^. Correlations between serum biomarkers of redox status and cartilage degradation and intervertebral disc disease and low back pain have not been described yet.

Also, whether LBP overall was associated with changes that might be masking smaller differences between patients with and without active discopathy remains unanswered. Values we found seemed different from the values reported in the general population. In previous studies, mean serum levels were 4.6 pg/ml for IL-1β, 18.3 pg/ml for IL-6, 38.3 pg/ml for IL-8, 37.0 pg/ml for TNF-α^22^, 291.6 (39.9) µmol/l for total thiols^23^, 125.8 (40.6) µmol/l for advanced oxidation protein products^23^, 0.3 (0.0) nM/mg of proteins for carbonyl groups^24^, 125.1 (3.7) nmol/l for Coll2-1 and 0.2 (0.1) nmol/l for Coll2-1NO2^25^. However, direct comparisons between previously published data and ours is not possible because blood samples were collected in heterogeneous populations, in an unstandardized manner and analyzed using different assays.

Even though inflammation is associated with disc activation, as suggested by the presence of infiltrated pro-inflammatory cells and the increased local expression of pro-inflammatory cytokines^26,27^, and may promote local redox status changes and cartilage degradation and the maintenance of an amplification loop between these 3 elementary molecular pathways^28^, one can hypothesize that local changes in inflammation, redox status or cartilage degradation are too low grade to translate into detectable serum biomarkers. Unfortunately, there is no synovial fluid in intervertebral disc as in peripheral joints^29^. One possibility could be to perform intervertebral disc biopsy but this procedure is too aggressive and not ethical.

Some other reasons may explain our findings: active discopathy was limited to a single disc, our sample size was small and not specifically calculated for the purpose of the present study, and our population was originally selected to address another question^12^. Therefore, our study may have lacked power and external validity. Because data about variability in the different measures was not available before our study, the possibilities to power our study properly were initially limited. Therefore, the present study should be considered as an exploratory study to identify biomarkers sensitive to the disease condition and to provide variability in the different measures. For example, based on our findings, if the true difference in the case and control means is 30 nM for Coll2-1 serum level with a standard deviation of 55, we would actually need to study 54 cases and 54 controls to be able to reject the null hypothesis with a power of 80% and a type I error probability of 0.05. Inevitably the statistical power will be different for the other measures for the same given sample size. As another important limitation, our findings pertain to only men with cLBP. In the primary study, we took several steps to exclude people with coincidental bone fragility, which resulted in the exclusion of women^12^. Whether we would have had similar findings in women is unknown.

## Conclusions

We did not find any differences in serum biomarkers between cLBP patients with and without active discopathy. Interpretation of our results is limited by the small sample size and the convenience sampling of our population. One plausible hypothesis is that local changes in elementary molecular pathways associated with active discopathy do not translate into detectable serum biomarkers.

## Data Availability

Full original protocol and dataset can be accessed upon request for academic researchers by contacting Associate Professor Christelle Nguyen (christelle.nguyen2@aphp.fr).
